# Taurine Administration Recovers Motor and Learning Deficits in an Angelman Syndrome Mouse Model

**DOI:** 10.3390/ijms19041088

**Published:** 2018-04-05

**Authors:** Sara Guzzetti, Luciano Calzari, Lucia Buccarello, Valentina Cesari, Ivan Toschi, Stefania Cattaldo, Alessandro Mauro, Francesca Pregnolato, Silvia Michela Mazzola, Silvia Russo

**Affiliations:** 1 Cytogenetics and Molecular Genetics Laboratory, Istituto Auxologico Italiano, IRCCS, 20145 Milano, Italy; guzzettisara@virgilio.it (S.G.); luciano.calza@gmail.com (L.C.); lucia.buccarello@marionegri.it (L.B.); 2 Department of Agricultural and Environmental Sciences, Università degli Studi di Milano, 20133 Milano, Italy; valentina.cesari@unimi.it (V.C.); ivan.toschi@unimi.it (I.T.); 3 Laboratory of Clinical Neurobiology, Istituto Auxologico Italiano, IRCCS, 28824 Piancavallo-Verbania, Italy; s.cattaldo@auxologico.it (S.C.); mauro@auxologico.it (A.M.); 4 Division of Neurology and Neurorehabilitation, Istituto Auxologico Italiano, IRCCS, 28824 Piancavallo-Verbania, Italy; 5 Department of Neurosciences, Università di Torino, 10126 Torino, Italy; 6 Experimental Laboratory of Immunological and Rheumatologic Researches, Istituto Auxologico Italiano, IRCCS, 20145 Milano, Italy; francesca.pregnolato@gmail.com; 7 Department of Veterinary Medicine, Università degli Studi di Milano, 20133 Milano, Italy; silvia.mazzola@unimi.it

**Keywords:** Angelman syndrome, taurine oral administration, motor and learning recover of deficit

## Abstract

Angelman syndrome (AS, MIM 105830) is a rare neurodevelopmental disorder affecting 1:10–20,000 children. Patients show moderate to severe intellectual disability, ataxia and absence of speech. Studies on both post-mortem AS human brains and mouse models revealed dysfunctions in the extra synaptic gamma-aminobutyric acid (GABA) receptors implicated in the pathogenesis. Taurine is a free intracellular sulfur-containing amino acid, abundant in brain, considered an inhibiting neurotransmitter with neuroprotective properties. As taurine acts as an agonist of GABA-A receptors, we aimed at investigating whether it might ameliorate AS symptoms. Since mice weaning, we orally administered 1 g/kg/day taurine in water to *Ube3a*-deficient mice. To test the improvement of motor and cognitive skills, Rotarod, Novel Object Recognition and Open Field tests were assayed at 7, 14, 21 and 30 weeks, while biochemical tests and amino acid dosages were carried out, respectively, by Western-blot and high-performance liquid chromatography (HPLC) on frozen whole brains. Treatment of *Ube3a^m^*^−/p+^ mice with taurine significantly improved motor and learning skills and restored the levels of the post-synaptic PSD-95 and pERK1/2-ERK1/2 ratio to wild type values. No side effects of taurine were observed. Our study indicates taurine administration as a potential therapy to ameliorate motor deficits and learning difficulties in AS.

## 1. Introduction

**Figure 1:**
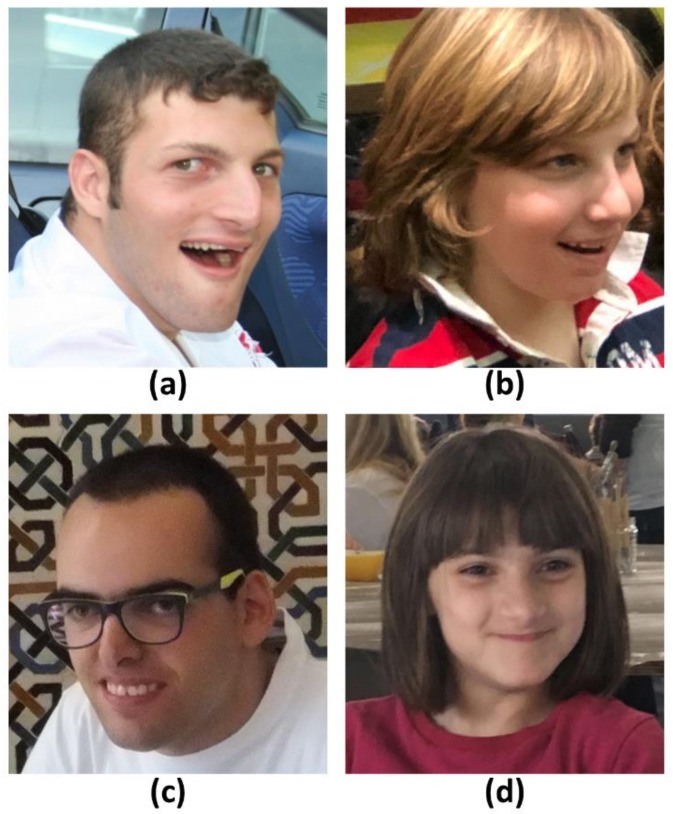
A photograph of AS patients representative of the genetic mechanisms: (**a**) deletion of 15q11–12; (**b**) chr.15 paternal uniparental disomy; (**c**) imprinting centre; (**d**) *UBE3A* point mutations. All patients’ parents have signed the informed consent.

Angelman syndrome (AS, MIM 105830) is a rare neurodevelopmental disorder affecting 1/10–20,000 children. The majority of AS patients share a moderate to severe development delay and intellectual disability, movement disorders and ataxia of gait, absence or limited speech and a peculiar behavior characterized by happy disposition, hyperexcitability and hand flapping, while over 80% of cases have relative microcephaly, seizures and a specifically altered EGG pattern with large-amplitude slow spike waves, sleep disorders [1,2]. Patients show a peculiar facies with prognathism, wide spaced teeth, pale skin and hair, addressing the clinical suspicion, but overall clinical manifestations are highly heterogeneous, partially depending on the specific molecular defects causing the disease. Figure 1 shows AS children at different ages and genetic defects evidencing the AS facies and its variability.

Genetic etiology consists of the lack of function of the maternal allele of *UBE3A* gene, mapping within the imprinted chromosomal region 15q11.2–q13 and coding for the E3 ubiquitin protein ligase involved in the ubiquitination of targeted proteins via proteasome degradation. Expressed in multiple cell compartment, UBE3A regulates oxidative metabolism in mitochondria, gene transcription in euchromatin-rich nuclear domains and might be involved in synaptic plasticity [3]. A paternally neuron specific long antisense transcript prevents the transcription of the paternal *UBE3A* allele, so in neurons, in spinal cord and peripheral nerves only *UBE3A* maternal allele is expressed [4]. Four molecular classes of defects impair *UBE3A* functionality: deletions encompassing the gene on the maternal allele (70% of cases), paternal uniparental disomy of chromosome 15q (3–5%), altered methylation at Imprinting Centre (3–5%) and point mutations within *UBE3A* coding sequence in up to 11%. *Ube3a* deficient mouse model [5] display phenotypes correlating with the human disorder, recapitulate features as motor deficits, learning and memory impairment, with differences depending on the strains. They greatly contributed to understand the molecular and cellular mechanisms involved in the pathophysiology of the disorder [6]. The potential dysfunction of GABAergic inhibitory system in Angelman syndrome has been investigated in animal model and in AS postmortem human brains revealing alterations that predict impairment of cortical extrasynaptic, but not synaptic GABAergic activity [7]. AS model mice also exhibit an increase in hippocampal mitochondrial reactive oxygen species which may contribute to the behavior deficit [8,9,10]. At the moment no efficacious therapy has been developed and the restoration of GABAergic neuronal function appears one of the most direct strategies to treat epileptic and neurological traits [11]. Administration of selective extrasynaptic GABA receptors agonist seems to ameliorate motor dysfunction in AS [12].

Taurine (2-β aminoethansulphonic acid) is a free intracellular sulfur-containing amino acid characterized by the presence of a sulfonate instead of a carboxyl group, not incorporated in proteins and representing the second most abundant amino acid in Central Nervous System (CNS) [13]. In human, taurine biosynthesis occurs primarily in the liver, but it may be consumed through food, mainly meat and seafood [14]. In CNS it exerts roles in cell volume regulation and in neurotransmission. Recently, various studies evidenced that taurine plays a neuroprotective role for CNS [15,16,17] and it is involved in neurogenesis [18]. Essential for the neuroprotection is its role in opening inhibitory anionic channels by activating glycine and/or non-synaptic GABA-A receptors [19]. Reyes-Haro et al. (2014) [20] showed taurine acts as weak agonist of GABA-A receptors in astrocytes and in STC-1 (Secretin tumor cell lines), cells expressing various GABA-A subunits.

On the basis of this evidence we designed the present study to investigate the efficacy of taurine administration to *Ube3a^m^*^−/p+^ mice in ameliorating their motor and learning dysfunction. Basing on reports showing that taurine, chronically administered both in food [21] or in drinking water [15,22,23] ameliorated motor and/or cognitive deficits of syndromic disorder, we orally administered 1 g/kg/day weight. A similar dosage has already been demonstrated successful and not toxic in a mouse model of Alzheimer’s disease [15]. Considering that the rescue of AS neurocognitive deficits achieved best results in an AS inducible mouse, when *UBE3A* was reactivated at three weeks [24], we chose to start taurine administration at the 21st day, just after weaning.

## 2. Results

Therapeutic effect of oral and chronic administration of taurine in motor impairment and learning difficulties of Angelman syndrome was examined administrating a battery of three behavioral tests to the transgenic *Ube3a^m^*^−/p+^ on the C57BL/6 background. Eight different experimental groups of animals were considered: untreated (1) male and (2) female *Ube3a^m^*^−/p+^; treated (3) male and (4) female *Ube3a^m^*^−/p+^; untreated (5) male and (6) female wild type *Ube3a^m^*^+/p+^ (wt) and treated (7) male and (8) female *Ube3a^m^*^+/p+^ wt.

### 2.1. Taurine Ameliorates Motor Coordination of Ube3a^m−/p+^ Mice

**Figure 2:**
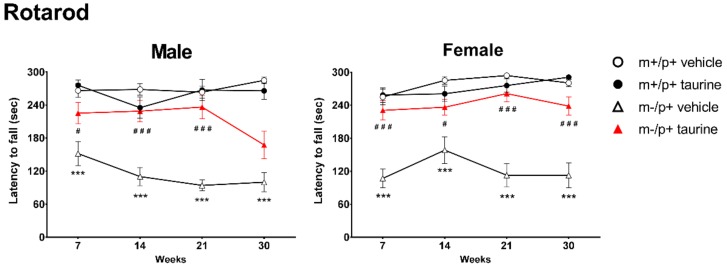
Taurine treatment improved motor coordination in AS (*Ube3a^m−/p+^*) mice. Rotarod tests were carried out at four time points (7, 14, 21 and 30 weeks) on wild type (wt) and *Ube3a^m−/p+^* mice (both treated (taurine) and untreated (vehicle)). Analyses were conducted by separating genders (male: **left panel**; female: **right panel**). Animals per group: male mice (wt + vehicle = 14, wt + taurine = 12, *Ube3a^m−/p+^* + vehicle = 11, *Ube3a^m−/p+^* + taurine = 11); female mice (wt + vehicle = 15, wt + taurine = 13, *Ube3a^m−/p+^* + vehicle = 12, *Ube3a^m−/p+^* + taurine = 14). Data are shown, for each time point, as media ± SEM. Effect of genotype: *** *p* < 0.001; effect of taurine treatment on *Ube3a^m−/p+^* genotype: ^#^ *p* < 0.05, ^###^ *p* < 0.001.

To identify differences in motor coordination, all the test groups were assayed through rotarod tests at four development time points: 7, 14, 21 and 30 weeks after birth. As showed in Figure 2, comparison between groups, untreated *Ube3a^m−/p+^* versus untreated wt mice, showed a significant impairment in rotarod performance (all time points: *** *p* < 0.001), while *Ube3a^m^*^−/p+^ mice administered with taurine since weaning showed a heterogeneous but significant amelioration. The only exception was observed for *Ube3a^m^*^−/p+^ male group at 30 weeks, showing no significant improvement. No significant effect was observed in controls after taurine treatment.

### 2.2. Open Field Test Reveals a Partial Improvement upon Taurine Administration

**Figure 3:**
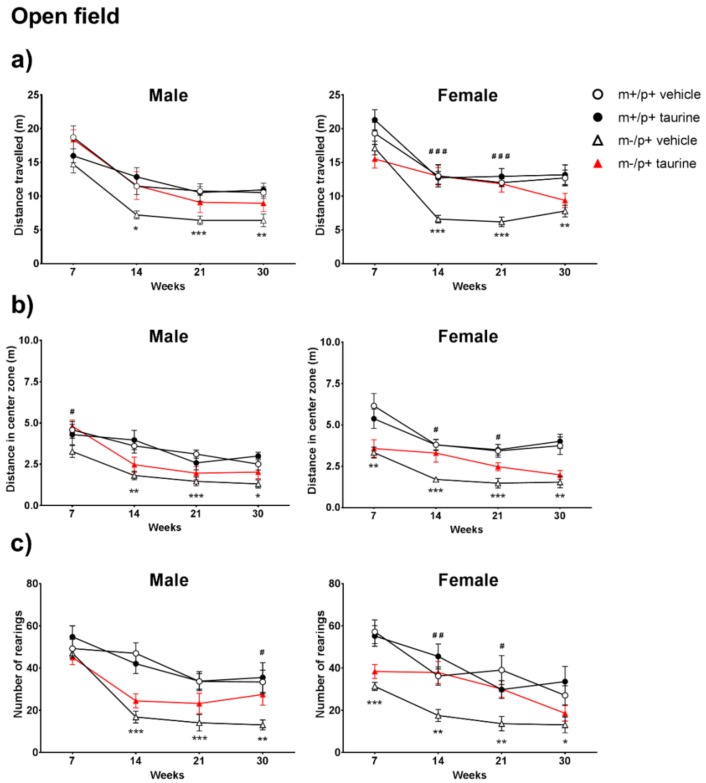
Taurine administration partially improved locomotor activity, anxiety and exploration in AS female (*Ube3a^m−/p+^*) mice. Open field assay was carried out at four time points (7, 14, 21 and 30 weeks) on wild type (WT) and *Ube3a^m−/p^^+^* mice (both treated (taurine) and untreated (vehicle)) by measuring (**a**) total distance traveled (m); (**b**) distance in center square (m) and (**c**) number of rearings. Analyses were conducted by separating genders (male: left panels; female: right panels). Number of animals per group: male mice (wt + vehicle = 13, wt + taurine = 12, *Ube3a^m−/p+^* + vehicle = 11, *Ube3a^m−/p+^* + taurine = 12); female mice (wt + vehicle = 13, wt + taurine = 13, *Ube3a^m−/p+^* + vehicle = 11, *Ube3a^m−/p+^* + taurine = 13). Data are shown, for each time point, as media ± SEM. Effect of genotype: * *p* < 0.05, ** *p* <0.01, *** *p* < 0.001; effect of taurine treatment on *Ube3a^m−/p+^* genotype: ^#^ *p* <0.05, ^##^ *p* <0.01, ^###^ *p* < 0.001.

The open field test (OFT) was used to evaluate locomotor activity, anxiety and exploration behavior in our groups: total distance, distance travelled in the center zone and number of rearings were then taken into account. Analysis of these parameters obtained by the ANY Maze software, indicated a behavioral deficit in the untreated *Ube3a^m−/p+^* versus untreated wt mice as revealed by the significant reduction of all observed OFT parameters (Figure 3). Since the 14th week, a significant decrease in total distance was observed (in both males and females) (Figure 3a—left and right panels), but it was more evident in females. Similarly, the significant differences in distance traveled in the central area as well as rearing number were more pronounced in females and became evident since the 7th week of life (Figure 3b,c—right panels).

Taurine treatment showed a beneficial effect mainly in female *Ube3a^m−/p+^* mice: a significant increase in locomotor activity was observed at the 14th and 21th week (^###^ *p* < 0.001) (Figure 3a—right panel). Taurine-treated *Ube3a^m−/p+^* males showed a partial but not significant rescue of locomotor performances (Figure 3a—left panel). Similarly, differences between genotypes in central area distances (Figure 3b—right panel) and rearing number (Figure 3c—right panel) were partially restored by taurine at the 14th and 21th week in *Ube3a^m−/p+^* female group (^#^ *p* < 0.05; ^##^ *p* < 0.01). A partial restore was also noted in males group, at the 7th week for central square distance (Figure 3b—left panel) and at the 30th week for rearing number (Figure 3c—left panel). For all parameters, no significant effect of taurine was observed in wt animals.

From a longitudinal view, a general flexion in measured parameters was observed in almost all test groups from the first time point (7th week) to other time points (14th and/or 21th and/or 30th week).

### 2.3. Learning and Memory Deficits Improve in Ube3a^m−/p+^ after Taurine Administration

**Figure 4:**
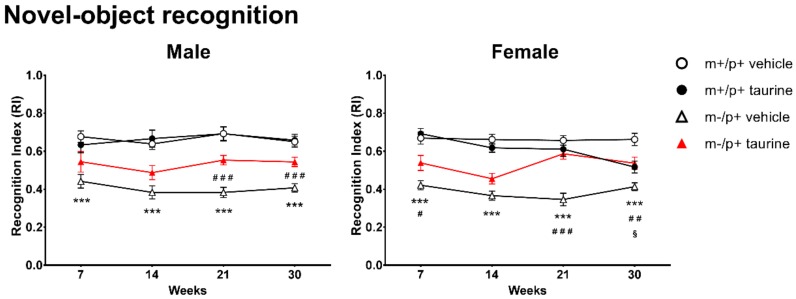
Taurine treatment improved learning and memory in AS (*Ube3a^m−/p+^*) mice. Novel object recognition test was carried out at four time points (7, 14, 21 and 30 weeks) on wild type (WT) and *Ube3a^m−/p+^* mice (both treated (taurine) and untreated (vehicle)). Analyses were conducted by separating genders (male: **left panel**; female: **right panel**). Number of animals per group: male mice (wt + vehicle = 14, wt + taurine = 11, *Ube3a^m−/p+^* + vehicle = 14, *Ube3a^m−/p+^* + taurine = 13); female mice (wt + vehicle = 12, wt + taurine = 14, *Ube3a^m−/p+^* + vehicle = 12, *Ube3a^m−/p+^* + taurine = 14). Data are shown, for each time point, as media ± SEM. Effect of genotype: *** *p* < 0.001; effect of taurine treatment on *Ube3a^m+/p+^* genotype: ^§^ *p* < 0.05; effect of taurine treatment on *Ube3a^m−/p+^* genotype: ^#^ *p* < 0.05, ^##^ *p* < 0.01, ^###^ *p* < 0.001.

Novel object recognition test (NORT) was used to measure visual recognition memory and anxiety level in *Ube3a^m−/p^*^+^ mice along with *Ube3a^m^*^+/p+^ controls. Mice show an innate and natural ability to explore unfamiliar objects: low levels of exploration may correlate with a state of anxiety. Locomotor activity should be carefully monitored but is not a critical element that can invalidate the effectiveness of the NORT [25]. Hyperdinamic and non-interacting mice have been discarded or retested. Evaluation of recognition index (RI) (Figure 4) revealed a strong genotype effect (*Ube3a^m−/p+^* vs. wild type) evidencing, for both males and females, at each time point, a very limited skill to explore the unfamiliar object (*** *p* < 0.001). *Ube3a^m−/p+^* mice administered with taurine ameliorated their propensity for exploration: *Ube3a^m−/p+^* male mice at the 21th and 30th weeks (^###^ *p* < 0.001); *Ube3a^m−/p+^* female mice at the 7th (^#^ *p* < 0.05), 21th (^###^ *p* < 0.001) and 30th (^#^ *p* < 0.01) weeks. Surprisingly, at the 30th week *Ube3a^m+/p+^* female mice manifested a slight and borderline decrease in novel object preference. Longitudinal analyses highlighted, for both genders, the maximum improvement in the 30th week.

### 2.4. Effect of Taurine on Synaptic Markers

**Figure 5:**
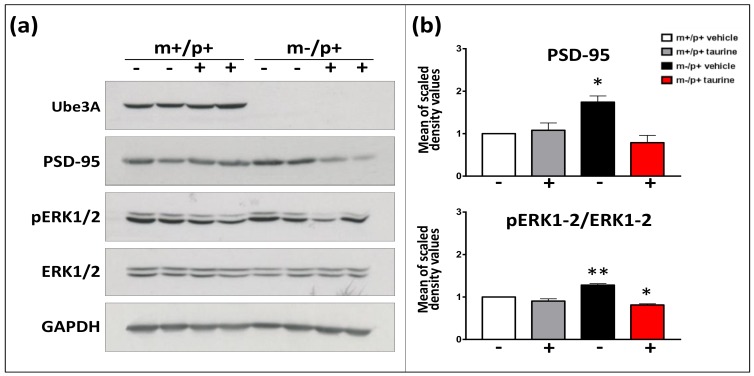
Activation of pERK1/2-ERK1/2 signaling and altered level of PSD-95 restored by taurine administration. Panel (**a**) Representative Western blots from female total brain homogenates show the expression of UBE3A (top blot), PSD-95, phosphorylated ERK1/2 and total ERK1/2 proteins, normalized by GADPH. m−/p+ (for *Ube3a^m−/p+^*) and m+/p+ (for *Ube3a^m+/p+^*) indicate the respective genotypes; the signs just below are referred to the treatment: + for taurine administration and—for vehicle. Panel (**b**) mean of scaled density values obtained from 3 independent experiments are showed. Significant differences between groups and untreated control are displayed with asterisks (* *p* < 0.05, ** *p* < 0.01).

To investigate if taurine administration may restore the synaptic deregulations pointed out by studies on AS patients [26,27,28], the level of the PSD-95, and the ratio between pERK1/2 and total ERK1/2 were investigated by Western blot in total brains from 30-week-old animals. As shown in Figure 5a,b, a slight increase of total PSD-95 (black bar) and activation of the ERK1/2 ratio (black bar) was found in transgenic mice. Taurine administration to transgenic mice restored values to wild type levels (red bar). Consequently, another member of MAPK signaling, transducing extracellular stimuli in intracellular cascade, JNK (c-Jun N-terminal kinase) was assayed showing a normal pJNK/JNK ratio in *Ube3a^m−/p+^* whole brain.

### 2.5. Taurine Content in Brain and Serum

**Figure 6:**
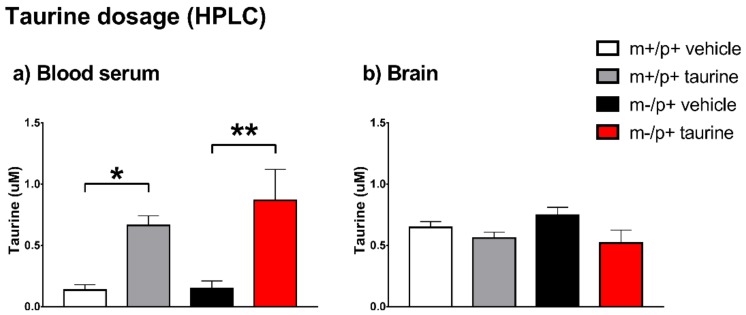
Blood serum Panel (**a**) and whole brain Panel (**b**) content of taurine evidenced a different accumulation in the two tissues. Measurement of taurine levels in blood serum and in whole brain of mice evaluated by high performance liquid chromatography. Asterisks indicate significant differences (* *p* < 0.05, ** *p* < 0.01). Number of mouse blood serums: wt + vehicle = 8, wt + taurine = 10, *Ube3a^m−/p+^* + vehicle = 6, *Ube3a^m−/p+^* + taurine = 6. Number of mouse brains: wt + vehicle = 13, wt + taurine = 8, *Ube3a^m−/p+^* + vehicle = 7, *Ube3a^m−/p+^* + taurine = 9.

**Figure A1:**
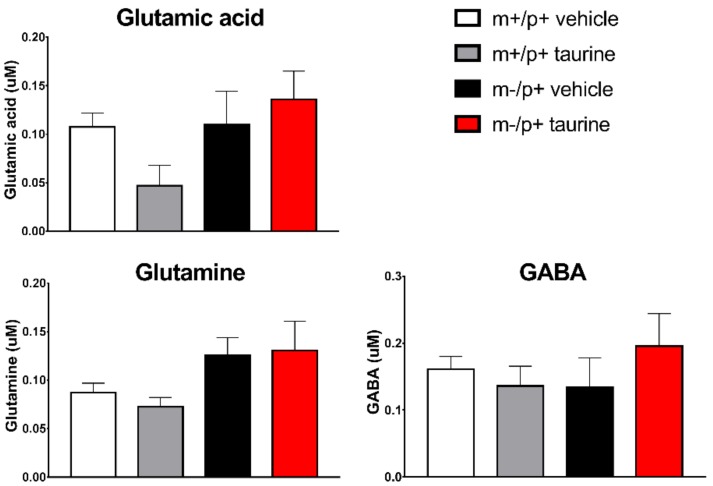
Measurements of neurotransmitters in whole brains evidenced no statistical differences between groups. HPLC experiments on whole mouse brain were carried out to quantify levels of Glutamic acid, Glutamine and Gamma AminoButyric Acid (GABA). Number of mouse brains: wt + vehicle = 13, wt + taurine = 3, *Ube3a^m−/p+^* + vehicle = 5, *Ube3a^m−/p+^* + taurine = 6.

We examined if the daily consumption of taurine in water altered the taurine content in serum and in whole brain. As shown in Figure 6, there were no differences in taurine level between untreated transgenic and wild type mice. A significant increase was observed in serum, where treated mice, independently from genotype, showed a taurine concentration 4–6-fold higher (Figure 6a). Conversely, the measurements of taurine in brain were similar in all tested groups (Figure 6b), according to the regulation of brain concentration by an active TauT transporter [16,29]. Quantification of additional neurotransmitters as Glutamic acid, GABA and Glutamine did not show significant differences (Appendix A, Figure A1).

## 3. Discussion

Angelman syndrome is a rare disorder severely affecting AS probands’ and their parents’ quality of life. Cognitive impairment, ataxic gait, absence of speech and occurrence of seizures are the main features of the syndrome that no therapy is available to ameliorate.

Our study reports that taurine dissolved in drinking water may restore motor deficits and learning impairment of a *Ube3a^m−/p+^* C57BL/6J mouse when administered daily, while the same treatment has no effect on motor and cognitive behavior of a wild type *Ube3a^m+/p+^* mouse. As the best period to obtain a rescue of behavioral deficits in the AS model seems to be the postnatal period [24], we started the treatment in the juvenile period of life (three weeks) as soon as the pups were able to feed by themselves. During the statistical evaluation, genders were considered separately to ensure a better homogeneity in the test groups and the amelioration appeared substantially overlapping, with the exception of the Open Field test. The rescue of motor deficit demonstrated by rotarod assay appeared since the first set of tests at the 7th week suggesting a strong effect of taurine on these skills; this point is supported by the performances at total distance walked in the OF test, a measure of ataxia attenuation, with significant improvement in the female group and a trend to improvement in males. A slight decrease of female performance at the last time-point may suggest that dosage could need to be adjusted during such a long chronic treatment. Variations among sex might be due to some environmental factors, as reported by Kovacs [30] in C57BL/6J mice. Memory and learning skills, as shown by NORT, results raised toward those of control animals, since the last two sets of evaluations, indicating the need of a longer treatment to achieve visible improvement of learning disability. Apparently, the prolonged administration of 1 g/Kg/day taurine did not reveal any toxicity [15]. Taurine is well known to increase neurogenesis of adult neural stem cells [18,31] and to promote neuroprotection preventing (i) glutamate-induced excitotoxicity through modulation of intracellular calcium homeostasis [32]; (ii) mitochondrial dysfunction by exerting an antioxidant activity; and (iii) apoptosis, by downregulating molecules that drive apoptotic events [16,17,33,34]. Potential beneficial effects of taurine in brain disorders, as Alzheimer’s [15], schizophrenia [16] and neurodevelopment disorder as fragile X syndrome have been reported [23,35]. Out of the multiple functions of taurine in the mammalian body, a key one is modulation of GABA neurotransmission. A defect in tonic GABAergic inhibition shared by fragile X and Angelman syndrome has been suggested to contribute to expression of the disease phenotype [11], raising the option to use agonists of GABA receptors as a winning strategy to restore the decrease in GABAergic inhibition [12]. Interestingly, taurine is considered an endogenous ligand of GABA extrasynaptic receptors [36]. Our study first investigates and proves the beneficial effect of taurine administration in ameliorating AS features of *Ube3a^m−/p+^* mice supporting the concept that GABA receptor agonist strategy may be effective. Neurological deficits in Angelman syndrome have been associated with an increased level of Arc (activity-regulated cytoskeletal-associated protein) which has the consequence to impair the Long Term Potentiation (LTP) and alter the recruitment of the scaffold protein PSD-95 (PostSynaptic Density-95), a marker of synaptic dysfunction [26]. Furthermore, UBE3A loss enhances the ERK1/2 (Extracellular Signal-Regulated Kinase) signaling pathway [27], which plays a relevant role in several biological processes including synaptic activity with a stimulatory effect on the proliferation of neural stem cells rather than in the following differentiation [37]. Our biochemical analyses confirmed the activation of ERK1/2 pathway and a major expression of the synaptic protein PSD-95 as a consequence of UBE3A deficit in the *Ube3a^m−/p+^* mice and demonstrated that taurine may restore a normal activity of both markers. We could speculate that the therapeutic effect of taurine in the AS model might be achieved for a restoration of both extrasynaptic GABAergic activity, with taurine being an agonist of the GABA receptor, and synaptic pathway as shown by our data on ERK1/2 activity and PSD-95. The dosage of taurine in the whole brain remained the same in treated and untreated animals while in serum we observed an a 4–6 folds increase. This finding is not completely unexpected considering the occurrence of active transport of taurine in brain, mediated by a specific saturable TauT transporter [16]. Sved D.W. et al. [29] investigated the absorption and tissue distribution when taurine was orally administered to rats using ^14^C taurine and observed that some tissues as brain, heart, muscles showed a slow increase of ^14^C taurine during the first 24 h and a small decrease during the following 168 h without changing the total amount of brain taurine. The authors supposed that after taurine passed the blood–brain barrier (BBB), it became part of an endogenous pool regulated by a Na^+^/Cl^−^ saturable transport within brain cells, which makes the taurine concentration in brain not proportional to plasma concentration. The same behavior has been described in muscles where despite a very high dosage of taurine being administered to a Duchenne dystrophy model, high performance liquid chromatography (HPLC) quantification revealed a higher concentration only in serum and in liver [38], while muscles maintained a constant concentration.

## 4. Materials and Methods

### 4.1. Animals

All subjects were heterozygous mice with deficiency of *Ube3a* on the maternal allele (*Ube3a^m−/p+^*) and wild type (*Ube3a^m+/p+^*) littermates, on the C57BL/6J background. Mice, developed by the Beaudet laboratory [39], were acquired from Charles River laboratories. Heterozygous *Ube3a^m−/p+^* females were crossed with wild type males (*Ube3a^m+/p+^*) to generate *Ube3a^m−/p+^* as well as wild type mice. The 21th day after birth, the animals were weaned and genotyped by PCR (from tail tissues), as described in [39]. Animals were bred with a regular 12:12 h light/dark cycle (lights on 07:00 a.m.), at a constant room temperature of 22 ± 2 °C, and relative humidity approximately 55 ± 10%. Animals were housed (*n* = 4 per group) in standard mouse cages. All mice were provisioned with bedding material (hard wood shavings), ad libitum food (Global Diet 2018S, Harlan Italy) and water. All procedures involving animals and their care were conducted in strict compliance with the national and international laws and policies and approved by the University of Study of Milano Animal Care and Use Committee (IACUC) (Approval Number: 95/14; Approval Date: 27 March 2014).

### 4.2. Tissue Collection

After behavioral experiments, mice at the 7th and 30th weeks were euthanized by cervical dislocation and the brains were immediately frozen in dry ice and stored at −80 °C prior to biochemical and immunohistochemical processing.

### 4.3. Taurine Administration

Taurine was orally administered via drinking water at dose level of 1000 mg/kg/day from weaning to 30 weeks of age. Taurine-based solution was prepared fresh twice a week adjusting volumes and amount of dissolved taurine for body weight changes and water intake Body weights were monitored twice a week for all the treated groups. To ensure the mice received the right dose required by the protocol, the effective amount of taurine intake was inspected every day for each cage. The animals did not suspend the taurine administration before tests, apart from the time they were trained or tested.

### 4.4. Test Groups

Four groups were considered for tests. Group 1: *Ube3a^m+/p+^* received drinking water only (control); Group 2: *Ube3a^m+/p+^* received taurine; Group 3: *Ube3a^m−/p+^* received drinking water only; Group 4: *Ube3a^m−/p+^* received taurine. Males and females were statistically analyzed separately.

### 4.5. Behavioral Tests

Tests were performed at 7th, 14th, 21th and 30th week of life. The equipment (e.g., open field arena) and all objects directly interacting with animals were cleaned using 70% ethyl alcohol to eliminate distracting/confounding olfactory stimuli.

#### 4.5.1. Rotarod

Mice were tested for balance and motor coordination by using an accelerating Rotarod (Ugo Basile 7650 model). Animals were initially trained for 4 sessions at a constant speed of 4 rpm: when properly trained they were tested twice (a 5 min-test) on a gradually accelerating rotarod (0.3 rpm/s increase from 3 to 300 rpm) by measuring the latency to fall from the bar (seconds). The final score was determined as the average of 2 trials.

#### 4.5.2. Open Field (OF) and Spontaneous Locomotor Activity

The open field test evaluates general locomotion activity, exploration behavior and level of anxiety by exposing mice to a novel and open space [40]. We used a 40 cm × 40 cm × 40 cm grey arena divided into 25 equal squares by black lines. During test, noises and lighting were consistently attenuated in order not to create artificial/external stress to mice. Mice were placed into the center of the floor defined as a “starting point” and allowed to explore for 5 min. Tests were video-recorded by a camera apparatus. Test parameters were obtained via the automated ANY-maze Behavior Tracking Software (Stoelting Co., Wood Dale, IL, USA). Total distance, distance in central square and number of rearings were taken into account.

#### 4.5.3. Novel-Object Recognition Test (NORT)

The novel-object recognition test (NORT) is a memory test relied on spontaneous animal behavior [41,42] aimed to exploit the natural exploration attitudes of mice. In the present study, tests were conducted in an open-field arena (40 cm × 40 cm × 40 cm) divided into 25 equal squares by black lines. The first day (day 1), animals were habituated for five minutes in a clean and empty arena: their movements were recorded as the number of line crossings, which provided an indication of both wild type and transgenic mice locomotion motor activities. In the next day (day 2), mice were re-placed in the same arena containing two identical objects (familiarization phase), randomly selected from a group of specific objects (a red rubber cylinder (4 cm × 5 cm), a plastic vial with a white cup (3 cm × 6 cm) and a plastic cube (3 cm × 5 cm)) to avoid bias among animals and between groups. Exploration was recorded in a 10 min trial by an investigator blinded to the genotype and treatment. In the familiarization phase, the time spent exploring the two identical objects (e.g., sniffing, touching, and stretching—at a minimum distance of 2 cm) was recorded and evaluated as object investigation. The test day (day 3), mice were still placed in the arena and exposed for 5 min to two different objects: one exposed during the familiarization phase, while the second one was something absolutely unfamiliar. As described above, time spent interacting with both objects was recorded and evaluated. A cut-off of 6 s was applied to the total exploration time (both during the familiarization and test phases): tests not meeting the minimum threshold were discarded. Recognition Index (RI), expressed as time spent exploring the novel object (TN) with respect to the total time spent exploring both objects (TN + TF) was estimated.

### 4.6. Western Blotting

Approximately 200 mg of brain tissue, were rapidly homogenized in ice-cold lysis buffer (Sucrose 0.32 M, Hepes 10 mM pH 7.4, PMSF 0.1 mM, NaHCO_3_ 1 mM, in presence of proteinase and phosphatase inhibitors, Complete Mini and PhosSTOP—Roche, Basel, Switzerland) using a mechanical homogenizer (Euro Turrax T20b, Ika Labor Technik, Staufen im Breisgau, Germany) at 27,000 rpm for 1 min, holding the sample on ice. Total homogenates were clarified by centrifugation at 1000× *g* for 5 min at 4 °C to remove crude nuclear material (P1). The supernatant (S1) was assayed for total protein content using a commercial protein assay kit according to manufacturer’s protocol (DC Protein Assay Kit—BioRad, Hercules, CA, USA).

Samples containing 30 μg proteins were resolved in 8% sodium dodecyl sulphate-polyacrylamide gels under reducing conditions and transferred to nitrocellulose membranes (iBlot Gel Transfer Stacks Nitrocellulose—Invitrogen, Carlsbad, CA, USA). Membranes were blocked for 1 h at room temperature in 5% milk solution, TBS (Tris-NaCl, pH 7.5) and tween 0.1% (TBT-T) and, then incubated with primary antibodies UBE3A (1:500; polyclonal; Proteintech Group, Rosemont, IL, USA), PSD-95 (1:1000; monoclonal; Millipore, Burlington, MA, USA), p44/42 MAPK (Erk1/2) (1:2000; polyclonal; Cell Signaling Technology, Danvers, MA, USA), Phospho-p44/42 MAPK (Erk1/2) (Thr202/Tyr204) (1:2000; monoclonal; Cell Signaling Technology), SAPK/JNK (1:1000; polyclonal; Cell Signaling Technology), Phospho-SAPK/JNK (1:1000; polyclonal; Cell Signaling Technology) and GAPDH (1:1500; polyclonal; Santa Cruz Biotechnology, Dallas, TX, USA) at 4 °C overnight in 5% milk TBS-T solution. Subsequently, membranes were washed and incubated for 1 h at room temperature in 5% milk, TBS-T solution with peroxidase-conjugated anti-rabbit or anti-mouse immunoglobulin G (Millipore) as secondary reagents. After washing with TBS-T, membranes were treated with ECL (Novex ECL Chemiluminescent Substrate Reagent Kit, Invitrogen). X-ray films were exposed in a linear range. Bands were quantified by using ImageJ software 1.8.0 (Available online: rsb.info.nih.gov/ij). Density value of each band was normalized against housekeeper protein GAPDH. Fold changes (with respect to control group) were calculated as the ratio of the average of normalized densities of each group (n per group = 2) and the average of the controls. Experiments were performed in triplicate.

### 4.7. HPLC Method

#### 4.7.1. Chemicals and Reagents

All chemicals and solvents were of analytical-reagent grade and used without further purification. Methanol HPLC grade was from VWR International, sodium acetate and 2-O-phthaldialdehyde was from Sigma-Aldrich (St. Louis, MO, USA).

#### 4.7.2. Samples Preparation

Brains were stored at −80 °C until analysis. Tissues were homogenized in 5 volumes (*w*/*v*) of methanol: 0.1 M Perchloric Acid 1:1 and then centrifuged for 5 min at 3000× *g* at 4 °C. 40 mL of supernatants were then used for derivatization procedure.

#### 4.7.3. Derivatization Procedure

The standards or samples were precolumn derivatized with 2-*O*-phthaldialdehyde (OPA) reagent solution. The derivatization reagent was freshly prepared every week and protected from light exposure, as described by Perucho in a previous study [43]. After derivatization, 35 µL of the mixture was injected into the analytical system.

#### 4.7.4. Chromatography System

An Agilent 1100 series HPLC system (Santa Clara, CA, USA) equipped with a Fluorescence Detector (flow-cell volume of 8 µL) was used for this study. The detector excitation and emission wavelengths were set at 240 and 450 nm, respectively. The Synergi Hydro-RP analytical column (80 Å, 150 mm × 2 mm × 4 µm) was used as stationary phase at ambient temperature [44]. Gradients were prepared with two degassed solvent mixtures. Solvent A was 0.05 M sodium acetate buffer, pH 5.88, and methanol (95:5), and Solvent B was methanol and double-distilled water (70:30). Gradient program was set as follows: initial conditions 25% B, linear step to 25% B from 0.1 to 3 min, gradient step to 33% B in 6.5 min, gradient step at 60% B for 2.5 min duration, jump to 25% B in 0.1 min; flow rate of 0.5 mL/min. Taurine concentration was calculated by comparison with calibrated external standard solution (1 µM).

### 4.8. Statistical Analyses

Statistical analyses were performed by using computing environment R and related specific “packages”. Graphics (plots and histograms) were generated by using GraphPad Prism (V.7) software. Values are presented as mean ± SEM. For all statistical approaches, a *p*-value < 0.05 was considered significant.

#### 4.8.1. Behavioral Tests

Data were analyzed by fitting General Estimating Equations (GEE) [45] using “geeglm” function (library “geepack”) with an AutoRegressive Order 1 (AR1) correlation structure and link = “identity”. Wald χ^2^ tests were conducted to evaluate the effect of genotype, treatment and time point (week) and their interactions. For each time point, individual group means were then compared using Tukey’s method to adjust *p*-values. For all behavioral tests, only mice with measurements in at least three time points were included in the analyses.

#### 4.8.2. Neurotransmitters Dosage

To evaluate differences in neurotransmitters (Taurine, Glutamic acid, Glutamine and GABA) levels in mouse brain and serum (only for taurine), inter-group comparisons were carried out by using two-way ANOVA, followed by TukeyHSD *post hoc* test.

#### 4.8.3. Western-Blot

Fold change differences with respect to untreated wild type group were analyzed by using a one-way ANOVA followed by TukeyHSD *post hoc* test.

## 5. Conclusions

This study first explores and proves the therapeutic effect of the sulfur-containing amino acid taurine, neurotransmitter agonist of GABAergic transmission involved in neurogenesis and neuroprotection, able to rescue the motor and learning deficit in a mouse model of the rare neurodevelopment disorder Angelman syndrome. The investigation of the involved mechanisms pointed to a restoration of the activation of the ERK pathway and of extrasynaptic GABAergic inhibition. The well-known role of taurine in preventing mitochondrial oxidative stress and inhibiting apoptosis as well as the proved enhancement of adult neural stem cells might also contribute to ameliorate the behavior deficits of AS model.

## Abbreviations

AS	Angelman Syndrome
UBE3A	Ubiquitin-protein ligase E3A
GABA	Gamma aminobutyric acid
CNS	Central nervous system
OFT	Open field test
NORT	Novel object recognition test
PSD-95	PostSynaptic Density-95
pERK1/2	Phospho-extracellular signal-regulated Kinase
ERK1/2	Total-extracellular signal-regulated Kinase
GAPDH	Glyceraldehyde-3-phosphate dehydrogenase
HPLC	High performance liquid chromatography
